# Non-Coding RNA Mediated Regulation of Allogeneic T Cell Responses After Hematopoietic Transplantation

**DOI:** 10.3389/fimmu.2018.01110

**Published:** 2018-06-15

**Authors:** Daniel Peltier, Pavan Reddy

**Affiliations:** ^1^Division of Hematology and Oncology, Department of Pediatrics, University of Michigan, Ann Arbor, MI, United States; ^2^Division of Hematology and Oncology, Department of Internal Medicine, Comprehensive Cancer Center, University of Michigan, Ann Arbor, MI, United States

**Keywords:** bone marrow transplantation, graft-versus-host disease, microRNA, T cell, alloimmunity

## Abstract

Allogeneic bone marrow transplantation (BMT) is an effective therapy for several malignant and non-malignant disorders. The precise control of allogeneic T cells is critical for successful outcomes after BMT. The mechanisms governing desirable (graft-versus-leukemia) versus undesirable (graft-versus-host disease) allogeneic responses remain incompletely understood. Non-coding RNAs (ncRNA) are controllers of gene expression that fine-tune cellular responses. Multiple microRNAs (miRNAs), a type of ncRNA, have recently been shown to influence allogeneic T cell responses in both murine models and clinically. Here, we review the role of various miRNAs that regulate T cell responses, either positively or negatively, to allo-stimulation and highlight their potential relevance as biomarkers and as therapeutic targets for improving outcomes after allogeneic BMT.

## Introduction

Donor-derived allogeneic T cells are the main effectors of both the curative graft-versus-leukemia/tumor (GVL) effect and the morbid and often mortal acute graft-versus-host disease (GVHD) following allo-bone marrow transplantation (BMT) (1). GVL and GVHD are alloimmune processes that are closely linked mechanistically and clinically, and the separation of the two remains the holy grail of allogeneic BMT (2–6).

Allogeneic T cells target allo-antigens on tumor cells and the main GVHD target organs (7). The process is initiated by host tissue damage secondary to cytoreductive conditioning regimens (1, 7, 8). Tissue damage from conditioning regimens promotes the release of inflammatory cytokines that activates antigen-presenting cells, which stimulate donor T cells with allo-antigens causing their expansion (1, 7, 8). In addition to expansion, alloreactive T cells differentiate into multiple helper T cell (CD4^+^) and effector T cell (CD8^+^) subtypes, all of which are involved in allogeneic T cell responses with Th1 and Th17 cells thought to promote GVHD, whereas Th2 and Treg cells limit GVHD (1, 7, 8). Following activation and differentiation, alloreactive T cells migrate to their target GVHD organs and/or tumor and cause damage/destruction *via* cell-mediated (e.g., *via* perforin/granzyme) or inflammatory cytokine (IFN-γ, TNF-α, and IL-1)-mediated processes (1, 7, 8). They also release cytokines and chemokines that promote the recruitment of mononuclear cells that aid in the final effector process. The cell-mediated allogeneic effector responses can be mediated by either the CD8^+^ cytotoxic T cells (CTLs) and/or aided by CD4^+^ T cells (1, 7, 8).

Non-coding RNAs (ncRNAs) lack protein-coding potential and are classified as small [<200 nucleotides (nt)] or long (>200 nt) ncRNAs. As evidence of their biologic and evolutionary importance, non-coding RNAs form the bulk of the transcribed mammalian genome, and organismal complexity better correlates with the fraction of the genome transcribed into ncRNA versus that transcribed into protein-coding genes (CDSs) (9, 10). There are many different types of small non-coding RNA, but microRNAs (miRNAs) are the most studied subtype contributing to gene regulation (11, 12). miRNAs are single-stranded and typically 19–22 nt in their mature form (11–13). Their nuclear precursors (pri-microRNAs) are transcribed *via* RNApol-II and processed by DROSHA to pre-microRNAs which are exported to the cytoplasm where they are cleaved by the endonuclease DICER to form mature miRNAs (11–13). Mature miRNAs associate with Argonaute family proteins to form RNA-induced silencing complexes that are then guided to specific mRNAs *via* base-pairing with its miRNA. One miRNA may target multiple genes, many miRNAs may target one gene, and the gene specificity of any given miRNA may vary depending on the cell type and context (12, 14–16). In T cells, miRNAs play important roles in T cell development, differentiation, activation, proliferation, survival, effector/regulatory functions, and immune reconstitution following allo-BMT; furthermore, multiple studies have shown crucial roles for miRNAs in the pathogenesis of hematologic malignancies and autoimmune disorders (17, 18). Consistent with their extensive role in T cell biology, ncRNAs, mainly miRNAs, have recently been shown to influence allogeneic T cell function and modulate aGVHD. In this review, we describe the emerging role of miRNAs on allogeneic T cell biology and discuss how many of these may prove to be useful biomarkers and therapeutic targets for aGVHD. In addition, we also describe the plausible role for another regulatory ncRNA, long non-coding RNAs (lncRNAs), in allogeneic T cells.

## Differential Expression of microRNAs in T Cells following Allo-Activation

The first analysis of miRNA differential expression in allogeneic T cells was carried out by Sun et al. (19), utilizing a novel global approach to identify differentially expressed miRNAs by co-immunoprecipitating Argonaut-bound miRNA and mRNA. The expression of these Argonaut-bound RNAs was then determined using microarrays (AGO-CLIP-CHIP). By comparing syngeneic, CD3/CD28-stimulated, and allogeneic *ex vivo* T cells from mixed lymphocyte reactions (MLRs), the authors identified a network of miRNAs that were dysregulated in the allogeneic samples relative to controls, including miR-142 which was subsequently confirmed *via* detailed studies reviewed below. The authors focused on miRNAs that were downregulated in the allogeneic T cells and showed that a group of mRNAs predicted to be targeted by these miRNAs also had a decreased enrichment following AGO-CLIP-CHIP. They confirmed these results utilizing *in vivo* murine models and further showed that the expression of several of the miRNAs predicted to target mRNAs was decreased as well.

Among these putative miRNA targets, the top two mRNAs regulated were the wings apart like homolog (*Wapal)* and synaptojanin 1 (*Synj1*). The study identified these as potentially novel proteins that regulate T cell biology. Wapal is a cohesin release factor that helps regulate chromatin architecture and is also required for sister chromatid resolution during mitotic prophase (20–22). The knockdown of Wapal caused T cells to proliferate slower only in response to an allogeneic stimulus. This intriguing finding suggests that Wapal may only play a minor role in T cell mitosis in response to a strong nonspecific stimulus, yet still be critical for regulating chromatin architecture and thereby gene expression in the context of alloimmunity. Synj1 is a known neuronal phosphatidylinositol phosphatase and a positive regulator of receptor-mediated endocytosis (20, 23–28). Its role in T cells, let alone allogeneic T cells, is unknown, but its role in neuronal synapse biology suggests that it may affect T cell vesicular trafficking, perhaps within the immunological synapse (29). When the expression of both Wapal and Synj1 was decreased *via* shRNAs, allogeneic T cells proliferated less and produced less inflammatory cytokines (IL-6, IL-17, and IFN-γ). Importantly, the effect on cytokine production was not global as IL-2 expression was preserved. Concurrent knockdown of Synj1 and Wapal in donor allogeneic T cells ameliorated recipient GVHD in mouse models. Nevertheless, the exact role and mechanism of Wapal and Synj1 in allo-T cell biology will need to be confirmed in T cell-specific genetic knockout models and in humans.

The differential expression of miRNAs in allogeneic T cells was also demonstrated by Jalapothu et al., utilizing an MHC-mismatched rat aGVHD model and the nanostring hybridization platform (16). Specifically, peripheral blood and intestinal T cells increased the expression of miR-99a, miR-223, miR-326, and miR345-5p. Importantly, the authors demonstrate a tissue-specific difference in miRNA expression and show that miR-146a and miR-155 increase in the skin following allo-BMT, which is similar to that discussed for T cells below. The differences in miRNA differential expression in allo-T cells between the Sun and Jalapothu studies likely reflect differences in technique, cellular source, and model systems.

## Experimental Data Demonstrating the Role for Specific miRNAs in GVHD and GVL

**Figure 1:**
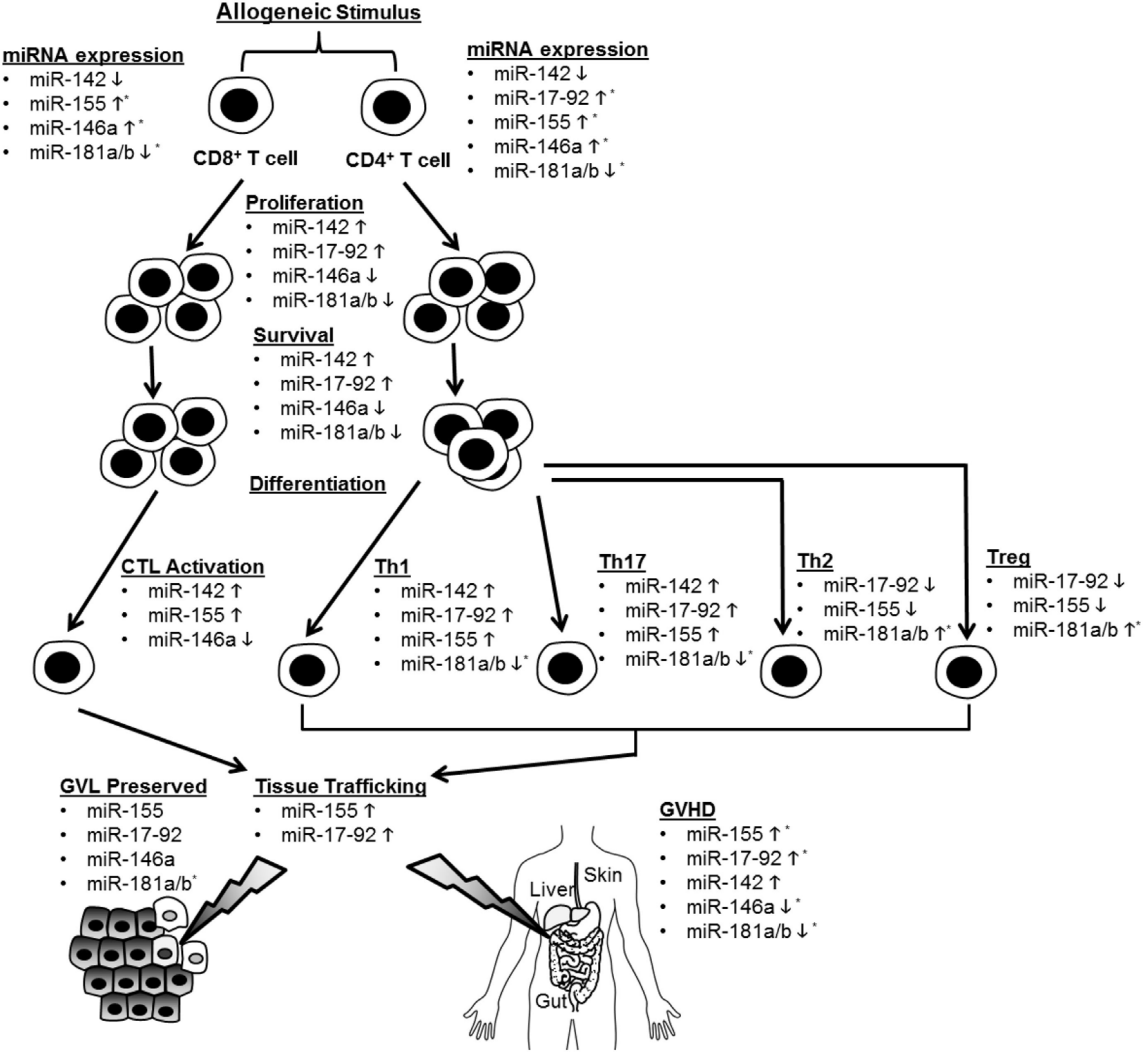
Summary of microRNA (miRNA)-mediated modulation of allogeneic T cell function. CD4^+^ and CD8^+^ T cells are depicted undergoing proliferation, survival, differentiation, tissue trafficking, and effector functions [graft-versus-leukemia/tumor (GVL) and graft-versus-host disease (GVHD)] following an allogeneic stimulus. miRNA expression in T cells is depicted as increased (5) or decreased (5) following allo-stimulation. The effect of individual miRNAs on the above aspects of allogeneic T cell biology is depicted as enhancing (5) or inhibitory (5). If an miRNA is not listed under a given allo-T cell function, its effect on this function was negligible or not tested. *Indicates that human data support this conclusion.

Recent studies have illuminated a role for several specific miRNAs in the regulation of T cell alloimmunity. They are shown in Figure 1 and Table 1.

**Table 1:** Influence of microRNA (miRNAs) on allogeneic T cells.

	miR-142	miR-17-92	miR-155	miR-146a	miR-181a/b
Change in expression following allo-stimulation	**↓**(54)	**↑**(45)^a^	**↑**(36, 40, 42–44)^a^	**↑**(43, 56, 60)^a^	**↓**(45, 61, 64)^a^
Effect on proliferation	**↑**(54)	**↑**(51)	N/A	**↓**(56)	**↓**(61)
Effect on survival	**↑**(54)	**↑**(51)	N/A	**↓**(56)	**↓**(61)
Effect on cytotoxic T cell activation	**↑**(54)	N/A	**↑**(36)	**↓**(56)	N/A
Effect on Th1 differentiation	**↑**(54)	**↑**(51)	**↑**(40)	N/A	**↓**(45)^a^
Effect on Th17 differentiation	**↑**(54)	**↑**(51)	**↑**(40)	N/A	**↓**(45)^a^
Effect on Th2 differentiation	N/A	**↓**(51)	**↓**(40)	N/A	**↑**(45)^a^
Effect on Treg differentiation	N/A	**↓**(51)	**↓**(40)	N/A	**↑**(45)^a^
Effect on tissue trafficking	N/A	**↑**(51)	N/A	N/A	N/A
Effect on graft-versus-leukemia/tumor	N/A	Preserved (51)	Preserved (36)	Preserved (56)	Preserved^a^ (45)
Effect on graft-versus-host disease	**↑**(54)	**↑**(51)^a^	**↑**(36, 40)^a^	**↓**(56, 59)^a^	**↓**(61)^a^
Potential biomarker	N/A	Yes^a^ (45)	Yes^a^ (43–45)	Yes^a^ (43, 45)	Yes^a^ (45, 64)

*N/A indicates a negligible effect or that it was not specifically tested*.

*^a^Indicates that human data support this conclusion*.

*References are in parentheses*.

### MicroRNA-155 (miR-155)

MicroRNA-155 is required for normal B and T cell function and known to enhance Th1 development and TNFα production (30–35). Ranganathan et al. extended miR-155’s functions to include the regulation of allogeneic T cells and promotion of GVHD (36). miR-155 expression was increased in both CD4- and CD8-positive cells following murine allo-BMT, and GVHD was ameliorated in multiple KO and antagomir-treated models and was exacerbated in over-expression models. Although the miR-155 KO had a reduced TNF expression, the authors showed no effect on alloreactive or homeostatic proliferation of T cells. miR-155 deficiency reduced the expression of chemokine receptors (CXCR4, CCR5, S1P1) required for leukocyte trafficking to GVHD target tissues. The authors also showed that IL-12RB1 and downstream components STAT4 and IFN-γ were reduced in miR-155-deficient allo-T cells. However, the specific and critical targets of miR-155 that are responsible for the effects on alloreactive T cells remain incompletely defined. Interestingly, antagomirs to miR-155 may also have beneficial antitumor effects (37–39).

Zhang et al. recently proposed that endothelial microparticles (EMPs) contribute to the increased miR-155 expression in allo-T cells (40). Purified EMPs from TNFα-stimulated endothelia cells were shown to transfer miR-155 to T cells. Interestingly, these EMPs enhanced *in vivo* GVHD, which was abrogated by EMPs generated from miR-155 antagomir-treated endothelial cells. However, the EMPs were added exogenously, which may not be relevant relative to the miR-155 produced within T cells. In addition to T cells, miR-155 deficiency in recipient dendritic cells was shown to ameliorate GVHD (reviewed elsewhere in this collection) making it a particularly attractive target for anti-GVHD therapeutics (41). Furthermore, elevated miR-155 levels were reported in lymphocytes from small and large intestine biopsies (*n* = 7) of GVHD patients relative to control biopsies (*n* = 3); miR-155 expression was increased following a bidirectional mismatched human MLR, and miR-155 showed promise as a peripheral blood GVHD biomarker (36, 42–45). Due to these encouraging data, a larger prospective clinic trial to assess if miR-155 expression can predict GVHD onset and severity is ongoing (NCT01521039).

### MicroRNA-17-92 Cluster

The microRNA-17-92 cluster encodes miR-17, 18a, 19a, 20a, 19b-1, and 92 and is known to be involved in tumorigenesis, B cell development/homeostasis, T cell differentiation, T cell survival, and T cell function (46–50). Its role in allo-BMT was explored, utilizing a T cell-specific KO model (51), which showed a reduced GVHD. The KO allo-T cells showed defects in proliferation, IFN-γ production, apoptosis, CXCR3 and α4β7 integrin expression, and allo-T cell recovery from the GI tract of hosts, all of which were consistently more pronounced in the CD4^+^ relative to the CD8^+^ compartment. By contrast, allogeneic CD8^+^ CTLs largely retained their cytolytic activity and retained sufficient GVL activity in these models. Antagomirs of either miR-17 or miR-19 protected the mice from GVHD with the miR-19 antagomir being slightly more efficacious. The seemingly minor effect of miR-17-92 on the allogeneic CD8^+^ compartment was somewhat unexpected given a previous report demonstrating miR-17-92’s role in the cytolytic activity and proliferation of CD8^+^ cells (48). Importantly, members of the miR-17-92 cluster were upregulated in plasma and CD4^+^ T cells from patients with aGVHD relative to those without (45). Further studies will need to be performed to identify the specific and critical targets of miR-17-92 responsible for the differential regulation of allogeneic CD4^+^ and CD8^+^ T cells as well as confirming these findings in human T cells.

### MicroRNA-142

MicroRNA-142 is expressed in hematopoietic cells, regulates leukocyte biology, and was one of the most decreased allogeneic T cell miRNAs following AGO-CHIP-CLIP discussed above (19, 52, 53). This prompted Sun et al. to examine its function in allogeneic T cells (54). The deletion of miR-142 in donor T cells protected mice from GVHD. MicroRNA-142-deficient allogeneic T cells were defective in proliferation, apoptosis, and production of inflammatory cytokines (IFN-γ and IL-17a). Unlike the microRNA-17-92 cluster, miR-142 deficiency appeared to effect the activation and proliferation of CD4- and CD8-positive cells equally. An antagomir to miR-142 protected mice from GVHD. However, it is unclear if the miR-142 antagomir would preserve GVL. To identify targets of miR-142, Sun et al. performed a microarray analysis on WT and KO T cells. Gene ontology analysis indicated that cell cycle-related functions were highly dysregulated in miR-142 KO T cells. Gene set function network prediction of upregulated genes in KO T cells showed that DNA replication functional genes were over-represented. Consistent with an increased expression of genes regulating cell cycle progression, miR-142 KO T cells demonstrated defective cell cycling characterized by S and G2/M phase arrest. The authors focused on the atypical E2F transcription factors, E2F7 and E2F8, which regulate the cell cycle and DNA replication (55) and are predicted targets of miR-142. These cell cycle defects were corrected by CRISPRi-mediated repression of E2F7/8. Furthermore, the GVHD-protective effect of miR-142-deficient donor T cells was abolished by CRISPRi-mediated repression of E2F7/8 in these cells, suggesting that E2F7/8 are novel targets for T cell alloimmune responses. Finally, the role of miR-142 in allo-T cells would be strengthened by human studies and verification by an independent group.

### MicroRNA-146a

MicroRNA-146a, in contrast to those miRs reviewed above, inhibits GVHD (56). MicroRNA-146a controls innate, adaptive, and autoimmunity (57). It targets TRAF6, which is required for the efficient activation of NFκB (58). Stickel et al. showed that miR-146a is induced in allo-T cells, and miR-146a^−/−^ T cells enhanced GVHD. The miR-146a^−/−^ T cells demonstrated enhanced proliferation, viability, and production of inflammatory cytokines (IL-6 and IFN-γ). MicroRNA-146a deficiency did not affect T cell differentiation, but enhanced TNFα production in allogeneic T cells. Consistent with this, TNFα blockade mitigated GVHD in miR-146a^−/−^ T cells. Transfection of donor T cells with a miR-146a mimic prior to transplantation ameliorated GVHD, as did intraperitoneal delivery of a miR-146a mimic starting 2 days post BMT. The anti-GVHD effect of miR-146a in mice was confirmed by an independent group (59). MicroRNA-146a also influences GVHD *via* its function in recipient dendritic cells (reviewed elsewhere in this collection) (60). Furthermore, the human SNP rs2910164, which inhibits miR-146a expression, showed a trend toward severe aGVHD risk, a finding confirmed in another publication (60). These clinical data will need to be validated in independent, larger cohorts; however, in support of miR-146a being relevant in human alloimmunity, the amount in peripheral blood may prove to be a useful GVHD biomarker (43, 45).

### MicroRNA-181a/b

The miR-181 family encompasses six miRNAs encoded by three paralogs (61). MicroRNA-181 inhibits negative regulators of T cell receptor signaling (62, 63). Two groups demonstrated that miR-181a in allogeneic T cells ameliorates GVHD (45, 61). Its expression is decreased in both plasma and CD4^+^ lymphocytes from patients with aGVHD, which correlated with the risk of aGVHD in a small cohort of patients and was confirmed as a potentially useful serum GVHD biomarker by an independent group (64). Human T cells transduced with a lentiviral vector overexpressing miR-181a revealed that miR-181a targets IFN-γ, inhibits Th1 and Th17 differentiation, enhances Th2 and Treg differentiation, inhibits proliferation, and enhances apoptosis. Despite murine and human miR-181a sequences being identical, when lentivirus overexpressing human miR-181a was injected into mice, there was no effect on IFN-γ expression in CD4^+^ T cells. Bioinformatics analysis revealed that the 3'UTR of murine IFN-γ was better matched for the seed sequence of miR-181b, which targets murine IFN-γ. Like miR-181a in humans, miR-181b levels were altered in the plasma of mice after the onset of GVHD, and allo-T cells transduced with a lentivirus overexpressing miR-181b reduced GVHD. In another study, transduced T cells overexpressing miR-181a and miR-181a/b-1^−/−^ donor T cells confirmed that T cell miR-181a regulates GVHD. Of note, both the study’s donor T cells were stimulated *ex vivo* to enhance transduction prior to BMT, which may have influenced outcomes. Regardlessly, further experiments will be required to definitively establish the mechanism and critical targets by which miR-181a/b in allo-T cells ameliorates GVHD.

## MicroRNAs as GVHD Biomarkers

MicroRNAs are increasingly being explored as noninvasive biomarkers in multiple diseases, including GVHD. Xiao et al. reported a panel of four plasma-derived miRNAs (miR-423, miR-199a-3p, miR-93, and miR-377) as a potential biomarker for the diagnosis, prognosis, and prediction of acute GVHD (65). The panel was based on the analysis of a small cohort of patients with GVHD relative to non-GVHD patients 6 weeks after BMT. MicroRNA expression was measured using RT-PCR-based microarrays and confirmed in a larger cohort. Of note, miR-155 and miR-30a were discarded from the final panel. When applied in a blinded fashion, this panel displayed 92% sensitivity for acute GVHD. Importantly, the prognostic and diagnostic utilities of these data were confirmed by Crossland et al. utilizing serum-derived miRNA from independent cohorts (66). Interestingly, Crossland et al. further demonstrate that extracellular vesicle-derived miR-423, miR-199, and miR-93 were lower in aGVHD patients at day + 14 relative to those who did not develop GVHD, which is the inverse expression pattern of these miRNAs in the serum (66). Another study utilized RT-PCR arrays to assess microRNA expression prospectively in 24 lymphoma patients undergoing matched unrelated donor HSCT (67). MicroRNA-194 and miR-518f were upregulated prior to and in acute GVHD samples. A recent study utilized a nanostring platform to characterize miRNAs in the serum of a diverse cohort of patients undergoing allo-BMT (68). At diagnosis of acute GVHD, miR-146a, miR-30b-5p, miR-374-5p, and miR-181a were downregulated and miR-20a and miR-15a were upregulated. Finally, several targeted studies demonstrated that miR-155, miR-146a, miR-146b, miR-153-3p, miR-181, miR-150, miR-17, miR-92a/b, and miR-586 may also serve as useful aGVHD biomarkers (43–45, 64, 69, 70).

Overall, these studies demonstrate great heterogeneity in the miRNAs identified as potential GVHD biomarkers. Several factors likely contributed to this heterogeneity including differences in patient populations, time points analyzed, type of body fluid analyzed, sample preparation, type of miRNA-profiling platform, spectrum of miRNAs profiled, and RNA normalization strategies (68). Regardlessly, these biomarker studies and the high-throughput studies conducted on murine allo-T cells detailed above highlight the numerous as yet uncharacterized miRNAs associated with alloimmunity.

## Long Non-Coding RNAs

Long non-coding RNAs are transcripts that regulate gene expression (10, 71). Like miRNAs, lncRNAs are transcribed in a manner analogous to mRNA except they are devoid of functional open reading frames (10). Unlike miRNAs, they are not processed by DICER/DROSHA pathways. They regulate gene expression in multiple ways, reviewed elsewhere (10).

The role lncRNAs play in allogeneic T cell responses is unknown. However, several observations suggest that this is plausible including (1) the expression of lncRNAs is specific for T cell subsets and differentiation (71–74) and (2) lncRNAs regulate other T cell functions known to be critical for alloimmunity including cytokine expression and migration (71–76). Nonetheless, it is unknown if lncRNAs influence allogeneic T cells. Like miRNAs, lncRNAs from body fluids and tissues are emerging as biomarkers, particularly for cancer diagnosis and prognosis (77). In addition, they may prove to be biomarkers and therapeutic targets for autoimmune disorders, inflammatory disorders (78, 79), and solid organ allograft rejection (80). Hence, it will be exciting to learn how lncRNAs influence allogeneic T cells and if they will be useful biomarkers or therapeutic targets for GVHD.

## Conclusion

Non-coding RNAs, particularly miRNAs, are emerging as important regulators of allogeneic T cells and may prove to be highly sensitive and specific biomarkers for GVHD. MicroRNAs enhance or inhibit GVHD *via* effects on multiple aspects of T cell biology, which is summarized in Figure 1 and Table 1. Several of these miRNAs demonstrate similar effects on allo-T cell biology, but the specific mRNA targets that are critical for regulating T cells remain to be explored for many of these miRNAs. Given the potential for multiple and different targets in distinct cell subsets, the effects of the miRNAs may be distinct in various T cell subsets. Nonetheless, the biological relevance of miRNA-mediated regulation is clear based on their unambiguous impact on GVHD with gain and loss of function experimental studies. Emerging data point to them serving as potentially useful biomarkers, although they will need to be validated in larger, prospective and better-controlled cohorts with standardized assays. While the biological mechanisms largely remain to be determined, these studies indicate that miRNAs may be useful targets for anti-GVHD oligonucleotide-based therapeutics. Although speculative, many of these oligonucleotides may even possess direct antitumor activity (e.g., antagomirs to miR-142, miR-155, and miR-17-92). These potential oligonucleotide therapeutics will need further investigation and rigorous study in formal clinical trials, including a heightened awareness of potential off-target effects and on-target toxicities such as immunosuppression. It is tempting to speculate that due to a greater tissue and disease-specific expression, lncRNA targeting may be more effective if they can be shown to regulate GVHD. Finally, a tailored cocktail of oligonucleotide medicines may demonstrate synergistic efficacy while mitigating side effects. In summary, ncRNAs are important regulators of allogeneic T cells, may be highly sensitive and specific biomarkers for GVHD, and might be useful targets for anti-GVHD medicines.

## Author Contributions

DP and PR wrote and edited the manuscript.

## Conflict of Interest Statement

The authors declare that the research was conducted in the absence of any commercial or financial relationships that could be construed as a potential conflict of interest.
